# A Review of Embodiment in Autism Spectrum Disorders

**DOI:** 10.3389/fpsyg.2013.00224

**Published:** 2013-04-30

**Authors:** Inge-Marie Eigsti

**Affiliations:** ^1^Department of Psychology, University of ConnecticutStorrs, CT, USA

**Keywords:** autism, ASD, embodiment, gesture, mimicry

## Abstract

In classical approaches to cognition, sensory, motor, and emotional experiences are stripped of domain-specific perceptual and sensorimotor information, and represented in a relatively abstract form. In contrast, the embodied cognition framework suggests that our representations retain the initial imprint of the manner in which information was acquired. In this paper, we argue that individuals with autism spectrum disorders (ASD) display impairments in the temporal coordination of motor and conceptual information (as shown in gesture research) and striking deficits in the interpersonal mimicry of motor behaviors (as shown in yawning research) – findings we believe are consistent with an embodied account of ASD that includes, but goes beyond, social experiences and is driven in part by significant but subtle motor deficits. In this paper, we review the research examining an embodied cognition account of ASD, and discuss its implications.

## What Does It Mean for Human Cognition to be Embodied?

Classic models of information processing in the cognitive sciences allow sensory, motor, and emotional experience to be represented as stripped of their perceptual and experiential basis. In such models, largely inspired by the metaphor of “mind as computer,” information taken in by the different sense modalities is preserved in memory in the form of abstract symbols, functionally separated from the original neural systems (those involved in motor action, vision, olfaction, and audition, for example) that encoded them in the first place.

In contrast, the theoretical framework of *embodied cognition* encompasses the notion that bodily experiences play an integral role in human cognition, and that our experiences are stored in a manner that maps onto the original neural systems (motor, visual, olfactory, and auditory) that encoded them in the first place. In this formulation, the ability to represent objects and events is subserved by sensorimotor systems that govern interactions with objects and events (Barsalou, 1999). When objects and events are recalled from memory to serve action goals, the sensorimotor systems involved in their initial representation are reactivated.

There are a number of research findings that are consistent with this hypothesis. For example, individuals hearing a description of a skyscraper tend to make vertical eye movements; those describing horizontally oriented structures tend to make side-to-side movements (Spivey et al., 2000). Similarly, individuals making judgment about events involving forward motion (“pushing closed a drawer”) respond more quickly if their response involves a similar motion (forward push) than an incompatible motion (e.g., responding to “opening the drawer” with a forward movement) (Glenberg and Kaschak, 2002). This suggests that envisioning the response has activated the motor schema, thereby facilitating (or interfering with) the subsequent response.

Embodied effects can be “offline,” as described above, or online, concurrent, effects (as described in Niedenthal et al., 2005). For example, in one classic study, individuals were asked to hold a pencil in their mouths while watching cartoons (Strack et al., 1988). In one condition, the pencil was oriented laterally, projecting away from the face; in a second condition, the pencil was oriented parallel to the mouth. These distinct facial positions were chosen because they activate, respectively, the musculature involve in frowning or smiling. The dependent measure in the study was participant judgments of cartoon humor; consistent with an embodied model of cognition, the “smile-activating” group rated the cartoons as significantly funnier. When subjects activated their smile musculature, as they watched a cartoon, they found the cartoon to be more humorous; the muscle activation influenced their representation of the cartoon. Effects were entirely implicit; although the relevant musculature was active, participants were not instructed to smile *per se*. There are a number of similar findings: when participants shake versus nod their heads (under the guise of judging the quality of headphones), while listening to a persuasive message, the listeners in the nodding condition are more likely to agree with the message in subsequent evaluation (Wells and Petty, 1980). Again, attitudes toward a stimulus are influenced by a physical (bodily) posture enacted when the individual encountered the stimulus; note that there is no *a priori* reason why smiling while seeing something should make one find that stimulus more humorous or pleasant, unless one is encoding the smile posture as part of the stimulus representation. The opposite is also true: when individuals’ motor movements are inhibited, there is *interference* in the experience of emotion and processing of emotional information (Niedenthal et al., 2005).

A construct that has played a role in the development of embodied approaches is the notion of “affordance perception” – that is, the qualities of objects that suggest to the perceiver *how* those objects are to be used (Gibson, 1977). For example, *how* does one know how to sit on a chair? Gibson (1977) proposed that qualities of the chair (its affordances) are independently available as percepts in the environment. We, as perceivers, make use of this information as we engage with the world. Research on children with Developmental Coordination Disorder suggests that these children are less aware of their own reaching capabilities (Johnson and Wade, 2009). Based on these findings, the authors hypothesize that impairments in skilled movements, generally, lead to differences in the ability to generate and detect information about affordances.

Casasanto and colleagues have formulated an *individual differences approach* to embodied cognition. Specifically, their body-specificity hypothesis proposes that people with “different kinds of bodies” think differently (Casasanto, 2011). For example, they show that right versus left-handed individuals represent abstract concepts differently, as revealed by their spontaneous gestures (Casasanto, 2009; Casasanto and Jasmin, 2010) and by differential patterns of brain activity (Casasanto, 2011). Certainly, this research would suggest that individuals with different motor control abilities are likely to exhibit differential cognitive representations; in this paper, we examine this possibility for the case of autism spectrum disorders (ASD).

There has been, to date, relatively little research directly exploring representation and cognition in individuals with ASD *from an embodied perspective*^1^. ASD refers to a developmental disorder characterized by atypicalities in three domains: social reciprocity; language and communication; and repetitive behavior and stereotyped interests. The three primary diagnoses that comprise the autism spectrum (Autistic disorder; Asperger’s syndrome; Pervasive Developmental Disorder, Not Otherwise Specified, PDD-NOS) share a similar pattern of deficits, though they differ in severity and long-term prognosis. Individuals with PDD-NOS typically exhibit better long-term outcomes, for example, than those with autistic disorder. The autism “spectrum” refers to variability across these diagnoses, as well as the large variability in IQ that can range from severe intellectual disability to the gifted range.

This research is valuable on at least two accounts (consistent with a framework proposed in the field of developmental psychopathology; Cicchetti and Rogosch, 1996). *First*, it holds the potential for revealing strengths and weaknesses in the embodied framework, by testing a population that presents with a wide range of abilities in the relevant domains. We propose that deficits in motor control and synchrony (at the cognitive and neural levels, reviewed below) have downstream effects on representation. As such, ASD may provide a useful test case for examining the framework of embodied cognition. *Second*, studies of ASD from an embodied perspective are likely to illuminate meaningful strengths and weaknesses, and potentially core deficits, in ASD. Klin et al. (2003) proposed that differences in embodied social cognition may cause a broad range of symptoms and differences in ASD. In this account, because individuals with ASD experience social stimuli as less salient (e.g., Dawson et al., 1998), seeking out and acting on physical rather than interpersonal stimuli, their experience of the world is “socially disembodied.” Theories of embodied cognition predict that experience with social interaction is, essentially, physically encoded; individuals who have less early experience with social contexts have reduced physical responses related to those contexts; their representations of the contexts, and subsequent physical responses in social contexts, are thus less automatic and efficient. This provocative hypothesis has received little empirical attention to date.

In this review, we provide a brief overview of embodied cognition; we next describe ASD, and then review the small set of studies that directly test embodiment effects in ASD. Based on the literature to date, we suggest that the role of embodiment in ASD may go beyond merely *social* contexts. Specifically, we propose that because motor processes are subtly impaired in ASD (reviewed in detail below), because of noisier synchronization of neural assemblies (Milne, 2011), or because of reduced cortical connectivity (Belmonte, 2004; Just et al., 2012), sensorimotor information may be *poorly integrated across modalities*. This leads in turn to impairments in the encoding of sensorimotor representations of the world; these noisier representations are more difficult to access and reproduce. We suggest that, because social representations are temporally evanescent, and complex, they are *more susceptible to noise*. In this account, individuals with ASD “embody” *all* their experiences differently, not just social ones; this difference impacts social along with other processes.

The notion of embodiment – that our sensorimotor input gives us access to the actions, emotions, and sensations, of other people (Gallese, 2006) – seems intuitively relevant to ASD, because individuals with ASD struggle to understand others, and to be as automatically and implicitly engaged with other people. We now review some potential “underpinnings” of embodiment, that may play an important role in the symptomatology of ASD.

## Motor Skills in ASD

If an individual cannot plan or implement a motor movement effectively, this will decrease the efficiency with which this person can build links between motor movements and other information (ideas, emotions, cognition, etc.). In other words, this individual will experience a different influence of embodiment. We turn now to a discussion of motor skills in ASD. Several decades ago, Rogers and Pennington (1991) proposed that motor skill deficits may be quite important in the symptomatology of ASD. This suggestion was seconded by a review of multiple studies (Smith and Bryson, 1994). More recently, Mostofsky has proposed that motor impairments are central in the phenotype of ASD (e.g., Mostofsky et al., 2006).

A variety of studies have shown motor coordination problems in ASD, including problems with: fine motor control (Szatmari et al., 1990); grip planning (Hughes, 1996); anticipatory movement preparation (Rinehart et al., 2001); gait and posture (Ghazziudin et al., 1992; Fournier et al., 2010), including shortened steps, “toe walking,” and generally poor coordination of limb movements (Vilensky et al., 1981); balance and coordination (Mari et al., 2003); imitation and pantomime (DeMyer et al., 1972; Stone et al., 1997); and reaching and grasping movements (Glazebrook et al., 2009).

Differences in motor skills may be present as early as infancy and toddlerhood (Teitelbaum et al., 1998; Brian et al., 2008; Dowell et al., 2009). For example, Teitelbaum et al. (1998) showed that early disturbances of movement (at 4–6 months) were apparent in home videos of infants later diagnosed with autism. Motor skills measured in children with ASD at age 5 years and again at age 7 years 11 months showed less improvement than in children with ADHD or non-specific developmental delays (Van Waelvelde et al., 2010). A study that followed a large (*n* = 95) group of toddlers longitudinally found that motor skills in infancy were a strong predictor of later social and communicative outcomes in ASD (Sutera et al., 2007) – *stronger* *than the severity* *of autism symptomatology*. Motor abilities are important predictors of outcomes in ASD.

Studies have consistently found that while planned actions may be ultimately “accurate” (e.g., individuals are able to reach for a target; see Dewey, 1993) in high-functioning autism, individuals with ASD are likely to have movements that exhibit significantly more temporal and spatial variability (Mostofsky et al., 2006, 2007, 2009; Glazebrook et al., 2009). Impairment often involves subtle anticipatory adjustments (Schmitz et al., 2003); for example, children with autism fail to anticipate the motor consequences of an action’s final goal (Cattaneo et al., 2007). Motor impairments in ASD can be subtle (Dowell et al., 2009) or absent; one elegant study of ball-throwing that required postural adjustment found absolutely no differences between ASD and control groups (Gidley Larson et al., 2008). Thus, the literature has been marked by what seem like highly inconsistent findings. These discrepancies were addressed in a recent review, which used a computational framework (rather than individual measures and tasks) to divide motor control into five components (Gowen and Hamilton, 2013). The authors concluded that findings were, in fact, consistent, showing impairments in ASD in two domains: (1) poor integration of information for efficient motor planning, and (2) deficits in organizing motor knowledge. They suggested that increased sensorimotor noise, and higher level motor planning, were both important contributors to ASD symptomatology. The issue of noisy representations as raised in the Introduction appears to have significant support from other domains.

There are clear parallels between the *production* of motion in ASD, and its *perception*, reflecting the relationship between perception and action more generally (Sperry, 1952). Many studies of motion perception have used a handy methodological tool known as “point-light displays,” a technique first described in Johansson (1973). In point-light displays, the participant sees a number of small, bright, dots on a dark background; the dots move in a coordinated fashion. Initially, the stimuli are created by fastening actual lights on the arms and legs of a moving person, dimming the lights, and then recording the resulting action; in this way, the body is invisible and the observer sees only the moving points of light. This is analogous to the real-life experience one might have of seeing only bobbing lights in motion, when a jogger clothed in black runs along a dark road wearing running shoes with reflective dots. Nowadays, stimuli are typically created using computer animation rather than actual lights.

While the many point-light display studies in ASD cannot be reviewed in detail here, one can draw several generalizations. Many studies of biological motion perception in ASD have reported striking differences in both behavioral and neural (especially fMRI) responses to such displays (Atkinson, 2009; Kaiser and Shiffrar, 2009; Nackaerts et al., 2012). These findings are not always replicated (McAleer et al., 2011), with differences reported for only *some* stimuli and tasks (Saygin et al., 2010). One potential resolution to the conflicting findings is that, when behavioral performance is carefully matched, individuals with ASD may activate clearly distinct brain networks as observed in fMRI (McKay et al., 2012). In other words, brain differences should only be interpreted for tasks on which behavioral performance is similar across groups (otherwise confounds of task difficulty/effortfulness render group differences less interpretable). If we assume that individuals with ASD do indeed exhibit impaired responses to point-light displays, this suggests that their perception of physical motion in other individuals is altered, which likely is reflected in their different mimicry abilities, reviewed next.

## Mimicry

Mental simulation refers to the process by which we compare a representation of someone else’s thoughts, feelings, or actions, to our own. This simulation could take place through the mediating mechanisms of mimicry and peripheral feedback. The ability to contrast our own with another person’s mental states has been described as a potentially critical impairment in ASD. *Mimicry*, likely one of the building blocks of mental simulation, involves the non-volitional, implicit, automatic matching of another person’s actions; it is clearly distinguished from imitation, defined as the deliberate, explicit, effortful reproduction of another person’s actions (Call and Tomasello, 1995). Many studies of typically developing (TD) individuals have demonstrated that when two people interact, an unconscious mimicry and synchronization of behavior occurs, impacting body posture, facial expressions, vocal prosody, speech patterns, emotions, and gestures (Niedenthal et al., 2005). Furthermore, the coupling of our automatic tendency to mimic and the effects of peripheral feedback on our inner emotional states may explain the phenomenon of emotional contagion (Hatfield et al., 1994). That is, because we unconsciously mimic the emotional movements of others, we unconsciously feel the emotions of others as we interact with them. The mimicry aspect of embodiment is so fundamental to our everyday interactions, that it is difficult to imagine being unaffected by the non-verbal signals and cues of those around us. It is likely responsible, at least in part, for why we enjoy comedic movies in a crowded movie theater more than we might enjoy such movies when watching at home alone. Research is still in the process of fleshing out the full cascade of consequences that might come about as the result of an early failure in mimicry. However, it seems likely that this failure would be catastrophic. Mimicry increases feelings of closeness and connection between individuals, which, in turn, increases the amount of mimicry individuals will display toward one another. One can imagine this process continuously unfolding in a cascade between caregiver and child throughout the early years of development. Abilities such as imitation, joint attention, and speech develop in the context of this increasingly synchronous bond. Strikingly, imitation (Smith and Bryson, 1994), joint attention (Kasari et al., 1990; Charman et al., 1997; Clifford and Dissanayake, 2008), and language (Eigsti et al., 2011; Mayo et al., 2013) are all signal impairments in ASD.

Research has suggested interactions between mimicry and communication. For example, one account describes conversation as “joint action” (Garrod and Pickering, 2004). A dialog between two interlocutors requires cooperation between those individuals in order for them to understand the meaning of the dialog; observers who do not participate are typically less accurate in their comprehension. The ease with which we engage in dialog, and our comprehension in dialogs, potentially involves *priming* of representations at multiple levels: phonological, lexical, syntactic, and semantic (Levelt and Kelter, 1982; Branigan et al., 2000; Hartsuiker et al., 2004). Priming reduces the effortfulness of conversation; it is likely that priming is similar to the notion of mimicry described above. Because individuals with ASD struggle with many aspects of conversation, including maintaining relevance, turntaking, task-switching between speaking and listening, higher level organization of narratives, and so on, it is possible that the model of interactive alignment provides a useful framework for understanding pragmatic and discourse impairments in ASD^2^.

Impairments in mimicry potentially underlie other social skills impairments in ASD. Adolescents and adults with high-functioning autism do not appear to exhibit normal automatic facial mimicry (McIntosh, 1996). Despite being able to produce typical facial expressions deliberately and explicitly, a group of individuals with ASD failed to exhibit mimicry of emotional expressions monitored via electromyography (EMG) (which monitors minute muscle contractions) when passively viewing emotional expressions. Similarly, while viewing emotional faces, adults with ASD had behaviorally intact performance, but decreased autonomic arousal, measured via galvanic skin conductance (Hubert et al., 2009). This suggests an altered implicit response to emotional faces. It should be noted that children with autism have been found to exhibit typical levels of autonomic arousal when viewing others in distress (Shenk and Ramachandran, 2003; Ben Shalom et al., 2006, November) even though children may not respond with typical *behaviors* to the distress of others (Sigman et al., 1992; Bacon et al., 1998). These studies suggest that when explicit and conscious behaviors are measured, responses to emotion displays may look intact; however, when we probe “under the hood” for more physiological responses, individuals with ASD look atypical. Clearly, for mimicry to be available as a source of feedback, an individual must attend to another person. Individuals with ASD, who often avoid looking at others, may be forced to rely solely on top-down cognitive strategies, rather then benefiting from bottom-up perceptually driven mimicry to understand other people’s emotions, ideas, and thoughts.

## Mimicry, Yawning, and Emotional Contagion

In our research, we have examined in detail one form of highly automatic mimicry: contagious yawning. Distinct from the spontaneous yawns that are observed in the human fetus, contagious yawns are prompted by seeing or hearing another person yawn. Sometimes yawning can be elicited by just reading the word; perhaps the readers of this chapter are, even now, stretching their jaws. Interestingly, susceptibility to contagious yawning is apparently associated with self-recognition and theory of mind, two abilities that contribute to complex empathy (Platek et al., 2003).

In our work, we tested contagious yawning in a large sample (*n* = 123) of TD children, ages 1–6 years, by reading a story to them for 12 min and deliberately yawning at four points during the reading (Helt et al., 2010). Results showed that TD children did not exhibit contagious yawning until age *four*. The late onset of contagious yawning implies that emotional contagion (a form of embodiment) becomes more developed and more sensitive over time, resulting in increased affective attunement with others. Furthermore, as part of the same study, we used the same method to elicit contagious yawning in a sample of 30 children with ASD ages 6–15 years (e.g., well beyond the point when TD children exhibit robust contagious yawning). Their responses were compared to a new group of chronological-age-matched (*n* = 28) or mental-age-matched (*n* = 28) TD children. In stark contrast to the TD participants, *none* of the children with autistic disorder and only three out of the 10 children diagnosed with PDD/NOS (milder ASD) yawned contagiously, as compared to 43% of an age-matched TD group. There was thus a relationship between diagnostic severity in ASD and susceptibility to contagious yawning. We hypothesized that the relationship reflected a difficulty in recognizing or acting on the correspondence between oneself and others, or a deficit in mimicking emotional behavior. When a person mimics (even unconsciously), the activation of emotional body schemas also creates the corresponding emotional reaction (i.e., the act of smiling causes us to feel happier, McIntosh, 1996), a phenomenon that may facilitate understanding the thoughts and feelings of others. Individuals with ASD may not experience emotional contagion during the early years of development.

Another study of implicit, spontaneous mimicry asked whether individuals with ASD are less likely to coordinate or synchronize their actions with a significant other. In this research (reviewed in Marsh et al., 2009), child-caregiver dyads were invited to sit in two adjacent rocking chairs of appropriate sizes, while the adult read aloud a book. During the reading, the adult was asked to rock her chair in tempo with a metronome that *only* the adult could hear. Analyses probed the relative synchrony of the child’s and adult’s rocking movements. Results indicated that the children with ASD were significantly less likely to coordinate their rocking with the caregiver, compared to a group of TD children and their caregivers.

A third study of non-conscious mimicry in ASD examined the kinematics of grasping objects as a tool for examining the relative impact on one person’s behaviors on another person’s action (Becchio et al., 2007). In this clever paradigm, children with autism and children with TD matched on age and gender (IQ was not assessed) watched a model reach out and grasp an object (in one condition), or look at an object (in another condition). Furthermore, on some trials, a second distracter object was present on the table. After the model completed the action (looking or grasping), the child was told to grasp the target object; in no case was there a distracter object in the participant’s display. Interestingly, in the TD group, participants’ reaches to objects where there had been a distracter for the model showed consistent kinematic differences, even though there was no distracter present. Effects were similar when the model simply *looked* at the target. In those cases, the child’s reach showed a less efficient path. In contrast, even though they looked as much at the model, the group with ASD showed no such difference. The findings suggested that the participants with autism were less influenced in their own actions by the actor’s gaze. This, and related, research depends on the underlying integrity of motor planning and control; that is, if motor control is impaired in ASD, as reviewed above, one would expect differences in all motor tasks; these differences may reflect not sociocommunicative impairments, but in reaching, grasping, and so on. In fact, in Becchio et al.’s (2007) reaching study, participants with ASD showed intact motor control in some conditions, but not others, allowing us to attribute performance impairments to differences in those conditions.

## Conversational Gestures and Embodiment in ASD

We turn now to a discussion of a phenomenon that we propose provides support for the possibility that even subtle differences in motor control in ASD have significant consequences, potentially reflecting a developmental difference in embodiment. Gestures – the spontaneous manual movements that accompany speech – are an important form of non-verbal communication that may facilitate early language learning and knowledge acquisition (McNeill, 1992). One category of gesture that is particularly relevant for the current paper is that of *iconic* gestures, which depict physical properties of referents. Iconic gestures often provide information that *complements* the information in the co-occurring speech. For example, a throwing motion can add information to the statement that “*he threw the coconut*,” for example by showing the direction (*over to the left*), or the manner (with *excitement* versus with *anger*) of the throwing action. Such gestures are informative about semantic representations.

Gestures undergo a similar developmental course as that of speech in language acquisition. In TD children, gestures precede first words (Bates et al., 1975) and may often substitute for specific lexical items (Acredolo and Goodwyn, 1988). Longitudinal studies have shown that children enter the first-word stage (at 10 months) producing more gestures than words (Capirci et al., 2005). The majority of objects to which children refer during this period are referred to first in gesture; they emerge in speech approximately 3 months later (Goldin-Meadow et al., 2007). Similarly, gesture-speech combinations (e.g., pointing to a hat while saying “dada”) emerge before two-word phrases (e.g., “dada hat,” McEacherns and Haynes, 2004; Özçaliskan and Goldin-Meadow, 2005; Goldin-Meadow et al., 2007). Gestures may facilitate early language development by offering an opportunity for symbolic representation *without* the complex motor sequences required by speech.

One influential theory proposes that gestures originate from the interface of speech and visuospatial thinking (Kita and Ozyurek, 2003); they are shaped by language and simultaneously express information that may not be encoded in speech (viz., visuospatial and motoric properties). Perhaps even more relevant to the current account is the “Gesture as Simulated Action” theory (Hostetter and Alibali, 2008). In this account, language (and gesture) involve simulations of perception and action that *activate or reactivate* perception and action states. Gesture specifically involves the simulation of motor and perceptual components of visuospatial imagery. In both theories, gestures encode visuospatial and motoric properties of lexical referents; in this role, gestures serve as the manifestation of action in a virtual environment. In other words, *conversational gestures reflect the operation of embodied cognition*, a notion supported by numerous empirical findings (Hanlon et al., 1990; Hansen et al. 2008; Iverson and Thelen, 1999; Kita and Ozyurek, 2003). Furthermore, hand gestures have a substantial impact on listeners, whose interpretations and subsequent movements are reliably affected by characteristics of speakers’ gestures (McNeill et al., 1994; Cook and Tanenhaus, 2009).

Gesture is thought to be specifically impaired in ASD, a fact reflected in the diagnostic criteria, in which gesture, and its integration with speech, is mentioned in numerous symptom criteria (American Psychiatric Association, 2000). The absence of pointing gestures (known as deictics) is considered an early warning sign of ASD; both the comprehension and production of deictics are found to be reduced (Mundy et al., 1986) and delayed (Camaioni et al., 1997) in ASD. Pointing is also often associated with joint attention, a major developmental milestone that involves sharing experiences with others (Mundy and Stella, 2000). Gestural joint attention skills are associated with language skills in children with ASD, such that children with reduced deictic use (for the purpose of drawing someone else’s attention to an object or event) are more delayed in early language acquisition (Loveland and Landry, 1986; Mundy et al., 1990; Bono et al., 2004). A reduced gestural repertoire has been observed in ASD (Wetherby and Prutting, 1984; Colgan et al., 2006), such that gestures fulfill fewer communicative functions.

Interestingly, most studies have failed to find group differences in *rates* of gesturing, after controlling for the amount of speech (Attwood et al., 1988; Capps et al., 1998). Rather than differences in gesture rate, or *quantity*, research and clinical description have both reported differences in gesture *quality*, including reduced synchrony with speech (Tantam et al., 1993), and “oddness” of greeting waves (Hobson and Lee, 1998). The unusual quality of gestures produced by individuals with ASD has long been noted in clinical accounts of the disorder. For example, Wing (1981) cites odd gestures in her case descriptions: “he uses large, jerky, inappropriate gestures to accompany speech” (Wing, 1981, p. 126). Similarly, Hans Asperger’s original account of the disorder noted the “large,” “clumsy,” and “inappropriate” gestures of the patients he described Asperger (1944), from Wing (1981). The odd quality of gestures, and their poor integration with speech, were noted by these influential clinicians.

Given these suggestive impressions that gesture quality may differ, our group examined the spontaneous gestures of adolescents with (*n* = 15) and without (*n* = 15) ASD, matched on age (ages 12–17), language level, and non-verbal IQ, as they told a story based on six cartoon prompts (de Marchena and Eigsti, 2010). There were striking group profiles. The adolescents with ASD produced as *many* gestures as their peers; there were large individual differences within each group, with some participants producing as few as two gestures during their story, and some producing as many as 23. However, their gestures were poorly *synchronized* with the semantically related speech. That is, in the ASD group, gestures were likely to either precede or follow the relevant speech by a lag of (on average) 333 ms. Furthermore, we asked raters (typical college students, entirely naïve to diagnosis and study questions) to judge the spontaneous narratives for clarity, how well the student could imagine the action, etc. Results indicated that the degree to which a narrative included poorly coordinated speech and gestures correlated strongly with ratings of communicative quality. It was also associated with ASD symptom severity. This kind of speech-gesture asynchrony appears to violate basic requirements for gestural comprehension (Habets et al., 2010), and it also seems to have devastating consequences for communication.

Gesture-speech coordination requires the efficient mobilization and ordering of distinct behaviors. There is a growing literature demonstrating impairments in behavioral timing in ASD. For example, electrophysiological studies have shown delayed responses to social stimuli by children with ASD, compared to TD peers (McPartland et al., 2004; Webb et al., 2006). A recent study of mimicry that measured facial muscle activity (via electromyography) showed that children with ASD differed from TD peers only in their *latency* to mimic (Oberman et al., 2009). Although the amount and appropriateness of mimicry was comparable, children with ASD took longer to mimic, suggesting that deficits in interpersonal synchrony were driven primarily by inefficient *timing* of behaviors, rather than by the *execution* of the behaviors themselves. Timing impairments are also present in non-social cognitive processes. For example, an intriguing study asked adults with and without ASD to complete an eyeblink conditioning procedure (Sears et al., 1994). In this task, a beep (tone) reliably precedes the delivery of a puff of air to the eye, eliciting an eyeblink. After training with tone-puff pairs, the individual receives a tone in isolation; when the individual blinks in response to the tone-alone stimulus, this is taken as evidence that the individual has learned the tone-puff association. In the Sears et al. (1994) study, the adults with ASD did show conditioned learning; however, their blink response was produced at a maladaptive interval, such that the eye reopened to its maximal aperture just as the puff of air arrived. Gesture deficits are also consistent with the hypothesis that individuals with ASD exhibit deficits in multi-modal sensory integration, as has been found for visual speech (lipreading) effects on auditory perception (Smith and Bennetto, 2007) and for temporal asynchrony between auditory and visual linguistic cues (Bebko et al., 2006).

Models of embodiment specify that the neural state that obtained when a stimulus was first encountered impacts subsequent processing of that stimulus. If timing impairments mean that the “initial state” is less clearly defined (e.g., that a given stimulus is not tightly coupled to the individual’s motor action), this should lead to relatively weaker embodiment effects. The above evidence of poor behavioral timing and synchrony in ASD is certainly consistent with this possibility. Furthermore, there is a relevant literature addressing the ease of performance of behaviors. Those behaviors that are performed frequently are well-learned and have a low threshold; in contrast, novel or very infrequent behaviors have a higher threshold. “Activation” refers to the relative strength of the behavior once the threshold is reached. A critical assumption is that the dynamic coupling of two systems – e.g., two limbs, or limbs and oral structures – requires relatively high levels of activation in order for mutual entrainment to occur. There is strong evidence of reduced information integration in ASD that is the direct consequence of decreased connectivity of local neural assemblies and of overconnectivity within local assemblies (Brock et al., 2002; Belmonte et al., 2004; Just et al., 2004, 2007; Rippon et al., 2007; Wicker et al., 2008). One suggestion from this literature is thus that, if an individual has activation that spreads less smoothly between brain regions (because of reduced connectivity, for example), that individual will be less able to mutually entrain a given system.

One strategy for addressing the question of whether “embodiment” is as strong an influence on individuals with ASD, is to examine directly embodied processes in that population. If a task were relatively non-social in nature, this would permit us to test specifically embodiment effects without the confound of whether an individual with ASD performs differently just because of a lifetime of reduced social interest and engagement. Although no such studies have been conducted to date, this research is in progress in our lab.

## Neural Mechanisms of ASD: Specific Differences in Embodiment

Some of the research on embodiment differences in ASD has been behavioral, but there are also several important pieces of evidence suggesting a solid neurophysiological foundation for these differences. Perhaps the most influential has been the documentation of reduced functional connectivity between distant brain regions in ASD (Belmonte, 2004; Just et al., 2004; Kana et al., 2006). Decreased connectivity between prefrontal and other cortical regions is specifically implicated in sociocognitive processing deficits in ASD (Wicker et al., 2008), and many researchers describe functional connectivity as tightly linked to these functional brain differences (Klin et al., 2003).

The cerebellum is involved in the timing and integration of behaviors, and has been shown to be anatomically atypical in ASD in multiple studies (Courchesne et al., 1988; Ritvo and Garber, 1988). Eyeblink conditioning, mediated by the cerebellum, requires rapid and precise timing, and is highly impaired in ASD (Sears et al., 1994), as described above. This autism-specific impairment is particularly striking, because 1- to 2-day-old newborns demonstrate eyeblink conditioning during sleep (Fifer et al., 2010); it is an early mastered ability. The cerebellum also controls the timing of behaviors that have both a cognitive and a motor component (Glickstein, 2006), and that require close synchrony (Katz and Steinmetz, 2002), such as speech production (Ackermann et al., 2004). Given all the differences in timing, temporal coordination, and synchrony, that seem to characterize ASD, and the importance of those processes for embodiment, researchers have looked carefully for specific links between cerebellar structure and function, and embodied processes.

## Mirror Neuron System and Embodiment in ASD

In addition to general discussions of functional connectivity and the cerebellum, many researchers have been excited by the prospect that the neural “architecture” underlying the mechanism of embodied cognition might be the mirror neuron system (MNS; Niedenthal, 2007; Gallese et al., 2013). The MNS refers to a set of neurons that fire when an action is *executed* and also when that same action is merely *observed*; as such, they appear to encode the observation and execution of action (Oztop and Arbib, 2002; Williams, 2008). Regions involved in MNS processing include the ventral part of the precentral gyrus, the posterior part of the inferior frontal gyrus, the rostral part of the inferior parietal lobe, and regions within the intraparietal sulcus and the superior temporal sulcus (Cattaneo and Rizzolatti, 2009). Mirror neurons have also been identified in supplementary motor area, an area mainly dedicated to movement initiation and sequencing, and medial temporal lobe, principally involved in memory tasks. Premotor areas active during the execution and the observation of an action may also be involved in the intention promoting the action (Gallese, 2006).

Research on the MNS suggests that social interaction draws on the capacity to predict and understand the motor goals and motor intentions of others, through their actions, and that this ability is instantiated in the cortical motor system organization via the MNS. It is possible that this network is established very early in development; studies of infants have suggested that there is “motor simulation” activity in premotor and posterior parietal cortex (Shimada and Hiraki, 2006). Mirror neurons respond most strongly to behaviors that are in our own behavioral repertoire (Buccino et al., 2004), implying that the more idiosyncratic a child’s emotional expressions and behaviors are, the less likely they may be to trigger mimicry in those around him.

While the MNS is an excellent candidate for serving as the neural substrate for embodiment, there is considerable controversy about its existence in humans and its specificity for understanding the symptomatology of ASD. While the original MNS data from primates are widely accepted, many researchers disagree about the existence and nature of the MNS system in humans. There is disagreement about the specific *location* of the mirror neurons; about whether there is a specific *interconnected system* of these neurons; and about whether there are *systematic differences* between neurons that perform mirroring functions, or whether any neuron can take on this function (e.g., Niedenthal, 2007). One review has suggested that the MNS hypothesis provides a useful model for understanding ASD, because “across the spectrum of autism-related disorders, it appears to be the cognitive functions that are embodied by action that are most affected” (Williams, 2008, p. 84). In sharp contrast, a more recent review of neuroimaging studies (Hamilton, 2013) concluded that studies using *non-emotional* hand action stimuli typically reveal *no group differences*, concluding that there is little evidence for global dysfunction of the mirror neuron system (Hamilton, 2013). More research is required to better understand the dynamics and anatomical substrates of the MNS in humans.

## Summary and Implications

While there have been no direct tests of embodied processes in ASD, we hypothesize that ASD is characterized by a relative decrease or lack of embodiment. That is, the stimuli that an individual with ASD encounters may be less bound to the sensory and motor conditions that held when that stimulus was first encountered. Because individuals with ASD seem to have motor deficits, involving poor integration of information for motor planning, and deficits in higher level motor planning, it seems possible that motor deficits contribute to a weakened role of embodied processing in functioning in individuals with ASD.

Language acquisition research provides evidence consistent with this possibility. For example, Linda Smith and colleagues have suggested an important role for sensorimotor functions in early word learning (Yu and Smith, 2012). That is, TD infants were most likely to learn words when the object to which a word referred was visually dominant, according to eyetracking data, and when they were physically manipulating those objects. Yu and Smith (2012) suggested that the infant’s visual focus on a specific object during naming, along with the infant’s handling of the object, served to reduce referential ambiguity. These behaviors were better predictors of the infant’s later word knowledge than was parent verbal labeling. Sensory-motor behaviors of infants and parents seemed to create optimal visual moments for learning, playing a stronger role in word learning than verbal naming by parents. These findings suggest that for language acquisition, sensorimotor cues help to constrain the learning process. If such constraints were not operating as efficiently in toddlers with ASD, due to motor control or sensorimotor integration deficits, one would anticipate language delays, as are found in ASD (Eigsti et al., 2007).

Given the pattern of findings described here, we suggest that there are (at least) three possible explanations for embodiment differences in ASD. Note that these explanations are not necessarily mutually exclusive.

1. The original encoding of stimulus information could be fuzzier. That is, information from the sensory and motor systems that encoded a stimulus may be less efficiently encoded in the relevant brain regions. This could reflect reduced connectivity, in that input from one sensory system (e.g., vision) is less synchronized with input from a second system (e.g., motor action or audition). In this case, the performance of individuals with ASD should be related in important ways to patterns of performance on learning and memory tasks.
2. Embodiment is, fundamentally, a motor output/motor planning problem. This seems possible given the many deficits in motor functioning reviewed above. For example, if the participants in embodied cognition paradigms are simply less effective at generating the appropriate physical postures, one would predict a reduced embodiment effect.
3. It is differences in attentional focus that govern the choice of experiences that are stored for later reactivation. Individuals with ASD are broadly less attentive to social, interpersonal, emotional information, and thus are likely to encode such information less accurately. Under this hypothesis, performance in ASD should improve for stimuli that are especially salient or of interest to an individual; this might be tested using stimuli for which the individual has a stereotyped or repetitive interest.

In general, it is of course true that development in TD individuals is shaped and sculpted by embodied processes. In particular, embodiment appears to be a particularly direct pathway for understanding the implications or associations of information. If individuals with ASD do not have access to this rich source of information, they must rely on alternative pathways to learn. Of course, in life, many important cues arrive via social interactions; this may explain why prior discussions of embodiment in ASD were limited to social cognition and social embodiment. The current data seem to suggest a broader reach of embodiment impairments in ASD.

These data raise several points to consider for intervention. Speaking generally, children with ASD benefit from explicit teaching of motor action and facilitating top-down mechanisms to bolster the less-active, implicit, bottom-up processes. These are processes that TD children use “for free” – that is, they engage bottom-up embodiment processes without explicit attention or effort. It is possible that explicitly directing children with ASD to adopt particular facial expressions or body postures, may help explicitly “entrain” the body with embodiment.

While data are limited, one study is consistent with this suggestion (Yilmaz et al., 2004). This intervention study involved teaching of a swimming intervention (“hydrotherapy”) to individuals with ASD. In addition to the benefits to physical health, including gains in balance, speed, agility, strength, flexibility, and endurance, this could potentially enhance motor control and motor planning and lead to downstream improvements in implicit mimicry, emotional contagion, and so on. Interventionists already engage in such explicit teaching of top-down approaches to skills in ASD, such as eye contact, that are absolutely critical in good social interaction. The current review also suggests that dyadic approaches that involve synchronization or mimicry training, perhaps via EMG, may also be a powerful approach.

While evidence of embodiment “impairments” in ASD is lacking, the collective impact of relevant studies reviewed here suggests that the time has come for a direct test of embodied processes in ASD.

## Conflict of Interest Statement

The authors declare that the research was conducted in the absence of any commercial or financial relationships that could be construed as a potential conflict of interest.

## Footnotes

^1^For example, a PubMed search for joint terms “embodied cognition” and “autism” yielded exactly 10 published papers, out of 22,138 for the terms independently (21,705 for “autism” and 433 for “embodied cognition”).

^2^We thank an anonymous reviewer for suggesting this potential link.

## References

[B1] Ackermann H. Mathiak K. Ivry R. B. (2004). Temporal organization of “internal speech” as a basis for cerebellar modulation of cognitive functions. Behav. Cogn. Neurosci. Rev. 3, 14–22 10.1177/1534582304263251 15191639

[B2] Acredolo L. Goodwyn S. (1988). Symbolic gesturing in normal infants. Child Dev. 59, 450–466 10.2307/1130324 2452052

[B3] American Psychiatric Association. (2000). Diagnostic and Statistical Manual of Mental Disorders IV-TR, 4th Edn Washington, DC: American Psychiatric Association

[B4] Asperger H. (1944). Die “autstichen Psychopathen” im Kindersalter. Arch. Psychiatr. Nervenkr. 117, 76–136 10.1007/BF01837709

[B5] Atkinson A. P. (2009). Impaired recognition of emotions from body movements is associated with elevated motion coherence thresholds in autism spectrum disorders. Neuropsychologia 47, 3023–3029 10.1016/j.neuropsychologia.2009.05.019 19500604

[B6] Attwood H. H. Frith U. Hermelin B. (1988). The understanding and use of interpersonal gestures by autistic and Down’s syndrome children. J. Autism Dev. Disord. 18, 241–257 10.1007/BF02211950 2970453

[B7] Bacon A. L. Fein D. Morris R. Waterhouse L. Allen D. (1998). The responses of autistic children to the distress of others. J. Autism Dev. Disord. 28, 129–142 10.1023/A:1026040615628 9586775

[B8] Barsalou L. W. (1999). Perceptual symbol systems. Behav. Brain Sci. 22, 577–609; discussion 610–660. 10.1017/S0140525X99002149 11301525

[B9] Bates E. Benigni L. Camaioni L. Volterra V. (1975). The acquisition of performatives prior to speech. Merrill Palmer Q. 21, 205–226

[B10] Bebko J. M. Weiss J. A. Demark J. L. Gomez P. (2006). Discrimination of temporal synchrony in intermodal events by children with autism and children with developmental disabilities without autism. J. Child Psychol. Psychiatry 47, 88–98 10.1111/j.1469-7610.2005.01443.x 16405645

[B11] Becchio C. Pierno A. Mari M. Lusher D. Castiello U. (2007). Motor contagion from gaze: the case of autism. Brain 130, 2401–2411 10.1093/brain/awm171 17711981

[B12] Belmonte A. (2004). Temporal averaging of turbulence-induced uncertainties on coherent power measurements. Opt. Express 12, 3770–3777 10.1364/OPEX.12.000168 19483909

[B13] Belmonte M. K. Allen G. Beckel-Mitchener A. Boulanger L. M. Carper R. A. Webb S. J. (2004). Autism and abnormal development of brain connectivity. J. Neurosci. 24, 9228–9231 10.1523/JNEUROSCI.3340-04.2004 15496656 PMC6730085

[B14] Ben Shalom D. Mostofsky S. H. Hazlett R. L. Goldberg M. C. Landa R. J. Faran Y. (2006). Normal physiological emotions but differences in expression of conscious feelings in children with high-functioning autism. J. Autism Dev. Disord. 36, 395–400 10.1007/s10803-006-0077-2 16565884

[B15] Bono M. A. Daley T. Sigman M. (2004). Relations among joint attention, amount of intervention and language gain in autism. J. Autism Dev. Disord. 34, 495–505 10.1007/s10803-004-2545-x 15628604

[B16] Branigan H. P. Pickering M. J. Cleland A. A. (2000). Syntactic co-ordination in dialogue. Cognition 75, B13–B25 10.1016/S0010-0277(99)00081-5 10771277

[B17] Brian J. Bryson S. E. Garon N. Roberts W. Smith I. M. Szatmari P. (2008). Clinical assessment of autism in high-risk 18-month-olds. Autism 12, 433–456 10.1177/1362361308094500 18805941

[B18] Brock J. Brown C. C. Boucher J. Rippon G. (2002). The temporal binding deficit hypothesis of autism. Dev. Psychopathol. 14, 209–224 10.1017/S0954579402002018 12030688

[B19] Buccino G. Vogt S. Ritzl A. Fink G. R. Zilles K. Freund H. J. (2004). Neural circuits underlying imitation learning of hand actions: an event-related fMRI study. Neuron 42, 323–334 10.1016/S0896-6273(04)00181-3 15091346

[B20] Call J. Tomasello M. (1995). Use of social information in the problem solving of orangutans (*Pongo pygmaeus*) and human children (*Homo sapiens*). J. Comp. Psychol. 109, 308–320 10.1037/0735-7036.109.3.308 7554827

[B21] Camaioni L. Perucchini P. Muratori F. Milone A. (1997). Brief report: a longitudinal examination of the communicative gestures deficit in young children with autism. J. Autism Dev. Disord. 27, 715–725 10.1023/A:1025858917000 9455730

[B22] Capirci O. Contaldo A. Caselli M. C. Volterra V. (2005). From action to language through gesture: a longitudinal perspective. Gesture 5, 155–177 10.1075/gest.5.1.12cap

[B23] Capps L. Kehres J. Sigman M. (1998). Conversational abilities among children with autism and children with developmental delays. Autism 2, 325–344 10.1177/1362361398024002

[B24] Casasanto D. (2009). Embodiment of abstract concepts: good and bad in right- and left-handers. [Research Support, Non-U.S. Gov’t]. J. Exp. Psychol. Gen. 138, 351–367 10.1037/a0015854 19653795

[B25] Casasanto D. (2011). Different bodies, different minds: the body specificity of language and thought. Curr. Dir. Psychol. Sci. 20, 378–383 10.1177/0963721411422058

[B26] Casasanto D. Jasmin K. (2010). Good and bad in the hands of politicians: spontaneous gestures during positive and negative speech. PLoS ONE 5:e11805 10.1371/journal.pone.0011805 20676371 PMC2911380

[B27] Cattaneo L. Fabbri-Destro M. Boria S. Pieraccini C. Monti A. Cossu G. (2007). Impairment of actions chains in autism and its possible role in intention understanding. Proc. Natl. Acad. Sci. U.S.A. 104, 17825–17830 10.1073/pnas.0706273104 17965234 PMC2077067

[B28] Cattaneo L. Rizzolatti G. (2009). The mirror neuron system. Arch. Neurol. 66, 557–560 10.1001/archneurol.2009.41 19433654

[B29] Charman T. Swettenham J. Baron-Cohen S. Cox A. Baird G. Drew A. (1997). Infants with autism: an investigation of empathy, pretend play, joint attention, and imitation. Dev. Psychol. 33, 781–789 10.1037/0012-1649.33.5.781 9300211

[B30] Cicchetti D. Rogosch F. (1996). Developmental pathways: diversity in process and outcome. Dev. Psychopathol. 8, 597–600 10.1017/S0954579400006933

[B31] Clifford S. M. Dissanayake C. (2008). The early development of joint attention in infants with autistic disorder using home video observations and parental interview. J. Autism Dev. Disord. 38, 791–805 10.1007/s10803-007-0444-7 17917803

[B32] Colgan S. E. Lanter E. McComish C. Watson L. R. Crais E. R. Baranek G. T. (2006). Analysis of social interaction gestures in infants with autism. Child Neuropsychol. 12, 307–319 10.1080/09297040600701360 16911975

[B33] Cook S. W. Tanenhaus M. K. (2009). Embodied communication: speakers’ gestures affect listeners’ actions. Cognition 113, 98–104 10.1016/j.cognition.2009.06.006 19682672 PMC2763957

[B34] Courchesne E. Yeung-Courchesne R. Press A. G. Hesselink J. R. Jernigan T. L. (1988). Hypoplasia of cerebellar lobules VI and VII in infantile autism. N. Engl. J. Med. 318, 1349–1354 10.1056/NEJM198805263182102 3367935

[B35] Dawson G. Meltzoff A. N. Osterling J. Rinaldi J. (1998). Neuropsychological correlates of early symptoms of autism. Child Dev. 69, 1276–1285 10.1111/j.1467-8624.1998.tb06211.x 9839415 PMC4084601

[B36] de Marchena A. Eigsti I. M. (2010). Conversational gestures in autism spectrum disorders: asynchrony but not decreased frequency. Autism Res. 3, 311–322 10.1002/aur.159 21182208

[B37] DeMyer M. K. Alpern G. D. Barton S. DeMyer W. E. Churchill D. W. Hingtgen J. N. (1972). Imitation in autistic, early schizophrenic, and non-psychotic subnormal children. J. Autism Child. Schizophr. 2, 264–287 10.1007/BF01538169 4678763

[B38] Dewey D. (1993). Error analysis of limb and orofacial praxis in children with developmental motor deficits. Brain Cogn. 23, 203–221 10.1006/brcg.1993.1055 7507333

[B39] Dowell L. R. Mahone E. M. Mostofsky S. H. (2009). Associations of postural knowledge and basic motor skill with dyspraxia in autism: implication for abnormalities in distributed connectivity and motor learning. Neuropsychology 23, 563–570 10.1037/a0015640 19702410 PMC2740626

[B40] Eigsti I. M. Bennetto L. Dadlani M. B. (2007). Beyond pragmatics: morphosyntactic development in autism. J. Autism Dev. Disord. 37, 1007–1023 10.1007/s10803-006-0239-2 17089196

[B41] Eigsti I. M. de Marchena A. B. Schuh J. M. Kelley E. (2011). Language acquisition in autism spectrum disorders: a developmental review. Res. Autism Spectr. Disord. 5, 681–691 10.1016/j.rasd.2010.09.001

[B42] Fifer W. P. Byrd D. L. Kaku M. Eigsti I. M. Isler J. R. Grose-Fifer J. (2010). Newborn infants learn during sleep. Proc. Natl. Acad. Sci. U.S.A. 107, 10320–10323 10.1073/pnas.1005061107 20479232 PMC2890482

[B43] Fournier K. A. Kimberg C. I. Radonovich K. J. Tillman M. D. Chow J. W. Lewis M. H. (2010). Decreased static and dynamic postural control in children with autism spectrum disorders. Gait Posture 32, 6–9 10.1016/j.gaitpost.2010.02.007 20400311 PMC2919314

[B44] Gallese V. (2006). Intentional attunement: a neurophysiological perspective on social cognition and its disruption in autism. Brain Res. 1079, 15–24 10.1016/j.brainres.2006.01.054 16680812

[B45] Gallese V. Rochat M. J. Berchio C. (2013). The mirror mechanism and its potential role in autism spectrum disorder. Dev. Med. Child Neurol. 55, 15–22 10.1111/dmcn.12067 22924341

[B46] Garrod S. Pickering M. J. (2004). Why is conversation so easy? Trends Cogn. Sci. (Regul. Ed.) 8, 8–11 10.1016/j.tics.2003.10.016 14697397

[B47] Ghazziudin M. Tsai L. Y. Ghaziuddin N. (1992). Brief report: a reappraisal of clumsiness as a diagnostic feature of Asperger syndrome. J. Autism Dev. Disord. 22, 651–656 10.1007/BF01046333 1483982

[B48] Gibson J. J. (1977). “The theory of affordances,” in Perceiving, Acting, and Knowing, eds Shaw R. Bransford J. (Hillsdale: Erlbaum), 127–143

[B49] Gidley Larson J. C. Bastian A. J. Donchin O. Shadmehr R. Mostofsky S. H. (2008). Acquisition of internal models of motor tasks in children with autism. Brain 131(Pt 11), 2894–2903 10.1093/brain/awn226 18819989 PMC2577807

[B50] Glazebrook C. Gonzalez D. Hansen S. Elliott D. (2009). The role of vision for online control of manual aiming movements in persons with autism spectrum disorders. Autism 13, 411–433 10.1177/1362361309105659 19535469

[B51] Glenberg A. M. Kaschak M. P. (2002). Grounding language in action. Psychon. Bull. Rev. 9, 558–565 10.3758/BF03196313 12412897

[B52] Glickstein M. (2006). Thinking about the cerebellum. Brain 129(Pt 2), 288–290 16434421 10.1093/brain/awh728

[B53] Goldin-Meadow S. Goodrich W. Sauer E. Iverson J. (2007). Young children use their hands to tell their mothers what to say. Dev. Sci. 10, 778–785 10.1111/j.1467-7687.2007.00636.x 17973795

[B54] Gowen E. Hamilton A. (2013). Motor abilities in autism: a review using a computational context. J. Autism Dev. Disord. 43, 323–344 10.1007/s10803-012-1574-0 22723127

[B55] Habets B. Kita S. Shao Z. Ozyurek A. Hagoort P. (2010). The role of synchrony and ambiguity in speech-gesture integration during comprehension. J. Cogn. Neurosci. 23, 1845–1854 10.1162/jocn.2010.21462 20201632

[B56] Hamilton A. F. (2013). Reflecting on the mirror neuron system in autism: a systematic review of current. Dev. Cogn. Neurosci. 3, 91–105 10.1016/j.dcn.2012.09.008 23245224 PMC6987721

[B57] Hanlon R. E. Brown J. W. Gerstman L. J. (1990). Enhancement of naming in nonfluent aphasia through gesture. Brain Lang. 38, 298–314 10.1016/0093-934X(90)90116-X 2322814

[B58] Hansen R. L. Ozonoff S. Krakowiak P. Angkustsiri K. Jones C. Deprey L. J. (2008). Regression in autism: prevalence and associated factors in the CHARGE Study. Ambul. Pediatr. 8, 25–31 10.1016/j.ambp.2007.08.006 18191778

[B59] Hartsuiker R. J. Pickering M. J. Veltkamp E. (2004). Is syntax separate or shared between languages? Cross-linguistic syntactic priming in Spanish-English bilinguals. [Comparative Study Research Support, Non-U.S. Gov’t]. Psychol. Sci. 15, 409–414 10.1111/j.0956-7976.2004.00693.x 15147495

[B60] Hatfield E. Cacioppo J. T. Rapson R. L. (1994). Emotional Contagion. New York: Cambridge University Press

[B61] Helt M. S. Eigsti I. M. Snyder P. J. Fein D. A. (2010). Contagious yawning in autistic and typical development. Child Dev. 81, 1620–1631 10.1111/j.1467-8624.2010.01495.x 20840244

[B62] Hobson R. P. Lee A. (1998). Hello and goodbye: a study of social engagement in autism. J. Autism Dev. Disord. 28, 117–127 10.1023/A:1026088531558 9586774

[B63] Hostetter A. B. Alibali M. W. (2008). Visible embodiment: gestures as simulated action. Psychon. Bull. Rev. 15, 495–514 10.3758/PBR.15.3.495 18567247

[B64] Hubert B. E. Wicker B. Monfardini E. Deruelle C. (2009). Electrodermal reactivity to emotion processing in adults with autistic spectrum disorders. Autism 13, 9–19 10.1177/1362361308091649 19176574

[B65] Hughes C. (1996). Brief report: planning problems in autism at the level of motor control. J. Autism Dev. Disord. 26, 99–107 10.1007/BF02276237 8819773

[B66] Iverson J. M. Thelen E. (1999). Hand, mouth & brain: the dynamic emergence of speech and gesture. J. Conscious. Stud. 6, 119–140

[B67] Johansson G. (1973). Visual perception of biological motion and a model for its analysis. Percept. Psychophys. 14, 201–211 10.3758/BF03212378

[B68] Johnson D. C. Wade M. G. (2009). Children at risk for developmental coordination disorder: judgement of changes in action capabilities. [Research Support, N.I.H., Extramural]. Dev. Med. Child Neurol. 51, 397–403 10.1111/j.1469-8749.2008.03174.x 19018835

[B69] Just M. A. Cherkassky V. L. Keller T. A. Minshew N. J. (2004). Cortical activation and synchronization during sentence comprehension in high-functioning autism: evidence of underconnectivity. Brain 127, 1811–1821 10.1093/brain/awh199 15215213

[B70] Just M. A. Cherkassky V. L. Keller T. A. Kana R. K. Minshew N. J. (2007). Functional and anatomical cortical underconnectivity in autism: evidence from an FMRI study of an executive function task and corpus callosum morphometry. Cereb. Cortex 17, 951–961 10.1093/cercor/bhl006 16772313 PMC4500121

[B71] Just M. A. Keller T. A. Malave V. L. Kana R. K. Varma S. (2012). Autism as a neural systems disorder: a theory of frontal-posterior underconnectivity. Neurosci. Biobehav. Rev. 36, 1292–1313 10.1016/j.neubiorev.2012.02.007 22353426 PMC3341852

[B72] Kaiser M. D. Shiffrar M. (2009). The visual perception of motion by observers with autism spectrum disorders: a review and synthesis. Psychon. Bull. Rev. 16, 761–777 10.3758/PBR.16.5.761 19815780

[B73] Kana R. K. Keller T. A. Cherkassky V. L. Minshew N. J. Just M. A. (2006). Sentence comprehension in autism: thinking in pictures with decreased functional connectivity. Brain 129, 2484–2493 10.1093/brain/awl164 16835247 PMC4500127

[B74] Kasari C. Sigman M. Mundy P. Yirmiya N. (1990). Affective sharing in the context of joint attention interactions of normal, autistic, and mentally retarded children. J. Autism Dev. Disord. 20, 87–100 10.1007/BF02206859 2139025

[B75] Katz D. B. Steinmetz J. E. (2002). Psychological functions of the cerebellum. Behav. Cogn. Neurosci. Rev. 1, 229–241 10.1177/1534582302001003004 17715595

[B76] Kita S. Ozyurek A. (2003). What does cross-linguistic variation in semantic coordination of speech and gesture reveal? Evidence for an interface representation of spatial thinking and speak. J. Mem. Lang. 48, 16–32 10.1016/S0749-596X(02)00505-3

[B77] Klin A. Jones W. Schultz R. Volkmar F. (2003). The enactive mind, or from actions to cognition: lessons from autism. Philos. Trans. R. Soc. Lond. B Biol. Sci. 358, 345–360 10.1098/rstb.2002.1202 12639332 PMC1693114

[B78] Levelt W. J. M. Kelter S. (1982). Surface form and memory in question answering. Cogn. Psychol. 14, 8–106 10.1016/0010-0285(82)90005-6

[B79] Loveland K. A. Landry S. H. (1986). Joint attention and language in autism and developmental language delay. J. Autism Dev. Disord. 16, 335–349 10.1007/BF01531663 3558291

[B80] Mari M. Castiello U. Marks D. Marraffa C. Prior M. (2003). The reach-to-grasp movement in children with autism spectrum disorder. Philos. Trans. R. Soc. Lond. B Biol. Sci. 358, 393–403 10.1098/rstb.2002.1205 12639336 PMC1693116

[B81] Marsh K. L. Richardson M. J. Schmidt R. (2009). Social connection through joint action and interpersonal coordination. Top. Cogn. Sci. 1, 320–339 10.1111/j.1756-8765.2009.01022.x 25164936

[B82] Mayo J. Chlebowski C. Fein D. A. Eigsti I. M. (2013). Age of first words predicts cognitive ability and adaptive skills in children with ASD. J. Autism Dev. Disord. 43, 253–264 10.1007/s10803-012-1558-0 22673858 PMC4386060

[B83] McAleer P. Kay J. W. Pollick F. E. Rutherford M. D. (2011). Intention perception in high functioning people with autism spectrum disorders using animacy displays derived from human actions. J. Autism Dev. Disord. 41, 1053–1063 10.1007/s10803-010-1145-1 21069445

[B84] McEacherns D. Haynes W. O. (2004). Gesture-speech combinations as a transition to multiword utterances. Am. J. Speech Lang. Pathol. 13, 227–235 10.1044/1058-0360(2004/024) 15339232

[B85] McIntosh D. N. (1996). Facial feedback hypothesis: evidence, implications, and directions. Motiv. Emot. 20, 121–147 10.1007/BF02253868

[B86] McKay L. S. Simmons D. R. McAleer P. Marjoram D. Piggot J. Pollick F. E. (2012). Do distinct atypical cortical networks process biological motion information in adults with autism spectrum disorders? Neuroimage 59, 1524–1533 10.1016/j.neuroimage.2011.08.033 21888982

[B87] McNeill D. (1992). Hand and Mind: What Gestures Reveal About Thought. Chicago, IL: University of Chicago Press

[B88] McNeill D. Cassell J. McCullough K. E. (1994). Communicative effects of speech-mismatched gestures. Res. Lang. Soc. Interact. 27, 223–237 10.1207/s15327973rlsi2703_4

[B89] McPartland J. Dawson G. Webb S. J. Panagiotides H. Carver L. J. (2004). Event-related brain potentials reveal anomalies in temporal processing of faces in autism spectrum disorder. J. Child. Psychol. Psychiatry 45, 1235–1245 10.1111/j.1469-7610.2004.00318.x 15335344

[B90] Milne E. (2011). Increased intra-participant variability in children with autistic spectrum disorders: evidence from single-trial analysis of evoked EEG. Front. Psychol. 2:51 10.3389/fpsyg.2011.00051 21716921 PMC3110871

[B91] Mostofsky S. H. Burgess M. P. Gidley Larson J. C. (2007). Increased motor cortex white matter volume predicts motor impairment in autism. Brain 130(Pt 8), 2117–2122 10.1093/brain/awm129 17575280

[B92] Mostofsky S. H. Dubey P. Jerath V. K. Jansiewicz E. M. Goldberg M. C. Denckla M. B. (2006). Developmental dyspraxia is not limited to imitation in children with autism spectrum disorders. J. Int. Neuropsychol. Soc. 12, 314–326 10.1017/S1355617706060437 16903124

[B93] Mostofsky S. H. Powell S. K. Simmonds D. J. Goldberg M. C. Caffo B. Pekar J. J. (2009). Decreased connectivity and cerebellar activity in autism during motor task performance. Brain 132(Pt 9), 2413–2425 10.1093/brain/awp088 19389870 PMC2732264

[B94] Mundy P. Sigman M. Kasari C. (1990). A longitudinal study of joint attention and language development in autistic children. J. Autism Dev. Disord. 20, 115–128 10.1007/BF02206861 2324051

[B95] Mundy P. Sigman M. Ungerer J. Sherman T. (1986). Defining the social deficits of autism: the contribution of non-verbal communication measures. J. Child Psychol. Psychiatry 27, 657–669 10.1111/j.1469-7610.1986.tb00190.x 3771682

[B96] Mundy P. Stella J. (2000). “Joint attention, social orienting, and nonverbal communication in autism,” in Autism Spectrum Disorders: A Transactional Developmental Perspective, eds Wetherby A. M. Prizant B. M. (Baltimore: Paul H. Brookes Publishing), 55–77

[B97] Nackaerts E. Wagemans J. Helsen W. Swinnen S. P. Wenderoth N. Alaerts K. (2012). Recognizing biological motion and emotions from point-light displays in autism spectrum disorders. PLoS ONE 7:e44473 10.1371/journal.pone.0044473 22970227 PMC3435310

[B98] Niedenthal P. M. (2007). Embodying emotion. Science 316, 1002–1005 10.1126/science.1136930 17510358

[B99] Niedenthal P. M. Barsalou L. W. Ric F. Krauth-Gruber S. (2005). “Embodiment in the acquisition and use of emotion knowledge,” in Emotion: Conscious and Unconscious, eds Barrett L. F. Niedenthal P. M. Winkielman P. (New York: Guilford), 21–50

[B100] Oberman L. M. Winkielman P. Ramachandran V. S. (2009). Slow echo: facial EMG evidence for the delay of spontaneous, but not voluntary, emotional mimicry in children with autism spectrum disorders. Dev. Sci. 12, 510–520 10.1111/j.1467-7687.2008.00796.x 19635079

[B101] Özçaliskan S. Goldin-Meadow S. (2005). Gesture is at the cutting edge of language development. Cognition 96, B101–B113 10.1016/j.cognition.2005.01.001 15996556

[B102] Oztop E. Arbib M. A. (2002). Schema design and implementation of the grasp-related mirror neuron system. Biol. Cybern. 87, 116–140 10.1007/s00422-002-0318-1 12181587

[B103] Platek S. Critton S. Myers T. Gallup G. (2003). Contagious yawning: the role of self-awareness and mental state attribution. Cogn. Brain Res. 17, 223–227 10.1016/S0926-6410(03)00109-5 12880893

[B104] Rinehart N. J. Bradshaw J. L. Brereton A. V. Tonge B. J. (2001). Movement preparation in high-functioning autism and Asperger disorder: a serial choice reaction time task involving motor reprogramming. J. Autism Dev. Disord. 31, 79–88 10.1023/A:1005617831035 11439757

[B105] Rippon G. Brock J. Brown C. Boucher J. (2007). Disordered connectivity in the autistic brain: challenges for the “new psychophysiology”. Int. J. Psychophysiol. 63, 164–172 10.1016/j.ijpsycho.2006.03.012 16820239

[B106] Ritvo E. R. Garber H. J. (1988). Cerebellar hypoplasia and autism. N. Engl. J. Med. 319, 1152 [Letter]. 10.1056/NEJM198810273191709 3173446

[B107] Rogers S. J. Pennington B. F. (1991). A theoretical approach to the deficits in infantile autism. Dev. Psychopathol. 3, 13–16 10.1017/S0954579400000043

[B108] Saygin A. P. Cook J. Blakemore S. J. (2010). Unaffected perceptual thresholds for biological and non-biological form-from-motion perception in autism spectrum conditions. PLoS ONE 5:e13491 10.1371/journal.pone.0013491 20976151 PMC2956672

[B109] Schmitz C. Martineau J. Barthelemy C. Assaiante C. (2003). Motor control and children with autism: deficit of anticipatory function? Neurosci. Lett. 348, 17–20 10.1016/S0304-3940(03)00644-X 12893415

[B110] Sears L. L. Finn P. R. Steinmetz J. E. (1994). Abnormal classical eye-blink conditioning in autism. J. Autism Dev. Disord. 24, 737–751 10.1007/BF02172283 7844097

[B111] Shenk L. M. Ramachandran V. S. (2003). Intact empathetic SCR in autistic individuals. Annual Meeting of the Society for Neuroscience, New Orleans, LA.

[B112] Shimada S. Hiraki K. (2006). Infant’s brain responses to live and televised action. Neuroimage 32, 930–939 10.1016/j.neuroimage.2006.03.044 16679032

[B113] Sigman M. D. Kasari C. Kwon J. H. Yirmiya N. (1992). Responses to the negative emotions of others by autistic, mentally retarded, and normal children. Child Dev. 63, 796–807 10.2307/1131234 1505241

[B114] Smith E. G. Bennetto L. (2007). Audiovisual speech integration and lipreading in autism. J. Child Psychol. Psychiatry 48, 813–821 10.1111/j.1469-7610.2007.01766.x 17683453

[B115] Smith I. M. Bryson S. E. (1994). Imitation and action in autism: a critical review. Psychol. Bull. 116, 259–273 10.1037/0033-2909.116.2.259 7526410

[B116] Sperry R. W. (1952). Neurology and the mind-body problem. Am. Sci. 40, 291–312

[B117] Spivey M. Tyler M. Richardson D. Young E. (2000). Eye movements during comprehension of spoken scene descriptions. Paper Presented at the 22nd Annual Conference of the Cognitive Science Society, Philadelphia

[B118] Stone W. L. Ousley O. Y. Littleford C. D. (1997). Motor imitation in young children with autism: what’s the object? J. Abnorm. Child Psychol. 25, 475–485 10.1023/A:1022685731726 9468108

[B119] Strack F. Martin L. L. Stepper S. (1988). Inhibiting and facilitating conditions of the human smile: a nonobtrusive test of the facial feedback hypothesis. J. Pers. Soc. Psychol. 54, 768–777 10.1037/0022-3514.54.5.768 3379579

[B120] Sutera S. Pandey J. Esser E. L. Rosenthal M. A. Wilson L. B. Barton M. (2007). Predictors of optimal outcome in toddlers diagnosed with autism spectrum disorders. J. Autism Dev. Disord. 37, 98–107 10.1007/s10803-006-0340-6 17206522

[B121] Szatmari P. Tuff L. Finlayson A. Bartolucci G. (1990). Asperger’s syndrome and autism: neurocognitive aspects. J. Am. Acad. Child Adolesc. Psychiatry 29, 130–136 10.1097/00004583-199001000-00021 2295566

[B122] Tantam D. Holmes D. Cordess C. (1993). Nonverbal expression in autism of Asperger type. J. Autism Dev. Disord. 23, 111–133 10.1007/BF01066422 8463192

[B123] Teitelbaum P. Teitelbaum O. Nye J. Fryman J. Maurer R. (1998). Movement analysis in infancy may be useful for early diagnosis of autism. Proc. Natl. Acad. Sci. U.S.A. 95, 13982–13987 10.1073/pnas.95.26.15688 9811912 PMC25000

[B124] Van Waelvelde H. Oostra A. Dewitte G. Van Den Broeck C. Jongmans M. (2010). Stability of motor problems in young children with or at risk of autism spectrum disorders, ADHD, and or developmental coordination disorder. Dev. Med. Child Neurol. 52, e174–e178 10.1111/j.1469-8749.2009.03382.x 20132135

[B125] Vilensky J. A. Damasio A. R. Maurer R. G. (1981). Gait disturbances in patients with autistic behavior: a preliminary study. Arch. Neurol. 38, 646–649 10.1001/archneur.1981.00510100074013 7295109

[B126] Webb S. J. Dawson G. Bernier R. Panagiotides H. (2006). ERP evidence of atypical face processing in young children with autism. J. Autism Dev. Disord. 36, 881–890 10.1007/s10803-006-0126-x 16897400 PMC2989721

[B127] Wells G. L. Petty R. E. (1980). The effects of overt head movement on persuasion: compatibility and incompatibility responses. Basic Appl. Soc. Psych. 1, 219–230 10.1207/s15324834basp0103_2

[B128] Wetherby A. M. Prutting C. A. (1984). Profiles of communicative and cognitive-social abilities in autistic children. J. Speech Hear. Res. 27, 364–377 6482406 10.1044/jshr.2703.364

[B129] Wicker B. Fonlupt P. Hubert B. Tardif C. Gepner B. Deruelle C. (2008). Abnormal cerebral effective connectivity during explicit emotional processing in adults with autism spectrum disorder. Soc. Cogn. Affect. Neurosci. 3, 135–143 10.1093/scan/nsn007 19015104 PMC2555468

[B130] Williams J. H. (2008). Self-other relations in social development and autism: multiple roles for mirror neurons and other brain bases. Autism Res. 1, 73–90 10.1002/aur.15 19360654

[B131] Wing L. (1981). Asperger’s syndrome: a clinical account. Psychol. Med. 11, 115–129 10.1017/S0033291700053447 7208735

[B132] Yilmaz I. Yanarda M. Birkan B. Bumin G. (2004). Effects of swimming training on physical fitness and water orientation in autism. Pediatr. Int. 46, 624–626 10.1111/j.1442-200x.2004.01938.x 15491399

[B133] Yu C. Smith L. B. (2012). Embodied attention and word learning by toddlers. Cognition 125, 244–262 10.1016/j.cognition.2012.06.016 22878116 PMC3829203

